# Rituximab in combination with cyclosporine and steroid pulse therapy for childhood-onset multidrug-resistant nephrotic syndrome: a multicenter single-arm clinical trial (JSKDC11 trial)

**DOI:** 10.1007/s10157-023-02431-0

**Published:** 2023-11-27

**Authors:** Kandai Nozu, Mayumi Sako, Seiji Tanaka, Yuji Kano, Yoko Ohwada, Tamaki Morohashi, Riku Hamada, Yasufumi Ohtsuka, Masafumi Oka, Koichi Kamei, Aya Inaba, Shuichi Ito, Tomoyuki Sakai, Hiroshi Kaito, Yuko Shima, Kenji Ishikura, Hidefumi Nakamura, Koichi Nakanishi, Tomoko Horinouchi, Akihide Konishi, Takashi Omori, Kazumoto Iijima

**Affiliations:** 1 Department of Pediatrics, Kobe University Graduate School of Medicine, 7-5-1 Kusunoki-Cho, Chuo-Ku, Kobe, 650-0017 Japan; 2 Division for Clinical Trials, Department of Clinical Research Promotion, Clinical Research Center, National Center for Child Health and Development, Tokyo, Japan; 3 Department of Pediatrics, Kurume University School of Medicine, Kurume, Japan; 4 Department of Pediatrics, Dokkyo Medical University School of Medicine, Tochigi, Japan; 5 Department of Pediatrics, Nihon University School of Medicine, Tokyo, Japan; 6 Department of Nephrology, Tokyo Metropolitan Children’s Medical Center, Tokyo, Japan; 7 Department of Pediatrics, Saga University School of Medicine, Saga, Japan; 8 Division of Nephrology and Rheumatology, National Center for Child Health and Development, Tokyo, Japan; 9 Department of Pediatrics, Graduate School of Medicine, Yokohama City University, Yokohama, Japan; 10 Department of Pediatrics, Shiga University of Medical Science, Shiga, Japan; 11 Department of Nephrology, Hyogo Prefectural Kobe Children’s Hospital, Kobe, Japan; 12 Department of Pediatrics, Wakayama Medical University, Wakayama, Japan; 13 Department of Pediatrics, Kitasato University School of Medicine, Kanagawa, Japan; 14 Clinical Research Center, National Center for Child Health and Development, Tokyo, Japan; 15 Department of Child Health and Welfare (Pediatrics), Graduate School of Medicine, University of the Ryukyus, Okinawa, Japan; 16 Clinical and Translational Research Center, Kobe University Hospital, Kobe, Japan

**Keywords:** Rituximab, Cyclosporine, MRNS, SRNS, Methylprednisolone

## Abstract

**Background:**

Only 80% of children with idiopathic nephrotic syndrome respond well to glucocorticoid therapy. Multidrug-resistant nephrotic syndrome (MRNS) is associated with a poor kidney prognosis. Several retrospective studies have identified rituximab as an effective treatment for MRNS; however, prospective studies are required to assess its efficacy and safety.

**Methods:**

We conducted a multicenter, non-blinded, single-arm trial to investigate the efficacy and safety of rituximab in patients with childhood-onset MRNS who were resistant to cyclosporine and more than three courses of steroid pulse therapy. The enrolled patients received four 375 mg/m^2^ doses of rituximab in combination with baseline cyclosporine and steroid pulse therapy. The primary endpoint was a > 50% reduction in the urinary protein/creatinine ratio from baseline on day 169. Complete and partial remissions were also evaluated.

**Results:**

Six patients with childhood-onset MRNS were enrolled. All patients were negative for pathogenic variants of podocyte-related genes. On day 169, five patients (83.3%) showed a > 50% reduction in the urinary protein/creatinine ratio, two patients showed partial remission, and two patients showed complete remission. No deaths occurred and severe adverse events occurred in two patients (infection in one patient and acute kidney injury in one patient). Three patients needed treatment for moderate-to-severe infection.

**Conclusions:**

The study treatment effectively reduced the urinary protein/creatinine ratio in patients with childhood-onset MRNS. The adverse events in this study were within the expected range; however, attention should be paid to the occurrence of infections.

**Supplementary Information:**

The online version contains supplementary material available at 10.1007/s10157-023-02431-0.

## Introduction

Childhood-onset nephrotic syndrome is common in early childhood (age 2–6 years) and is often associated with edema of the eyelids and lower legs at onset. The disease is idiopathic in approximately 90% of cases. The first-line treatment for childhood-onset idiopathic nephrotic syndrome is an oral glucocorticoid [1, 2]. Responsiveness to a glucocorticoid is an important factor in determining the course of treatment [3]. In 80–90% of patients, childhood-onset idiopathic nephrotic syndrome promptly resolves with glucocorticoid treatment (i.e., steroid-sensitive nephrotic syndrome); however, 80% of patients relapse, half of whom develop frequently relapsing or steroid-dependent nephrotic syndrome [4, 5]. Immunosuppressive therapy is indicated for such cases. Conversely, the remaining 10–20% of patients do not achieve remission within 4–8 weeks of glucocorticoid treatment (i.e., steroid-resistant nephrotic syndrome [SRNS]) [4]. According to a Cochrane review, cyclosporine is an effective induction therapy for glucocorticoid-resistant nephrotic syndrome [6]. Several cohort studies have also demonstrated the efficacy of steroid pulse therapy [7, 8]. In a cohort study targeting seven patients with childhood-onset SRNS with pathologically focal segmental glomerulosclerosis, a combination of five courses of steroid pulse therapy followed by 1 year of oral glucocorticoid therapy and 2 years of cyclosporine treatment resulted in a high remission rate (85.7%, six of seven cases) [9]. However, patients who do not achieve remission with such treatment, although an extremely rare condition, are considered to have multidrug-resistant nephrotic syndrome (MRNS) and progress to chronic kidney disease (CKD) stage 5 [10]. Progression to CKD stage 5 has been reported in approximately 40% of these cases [11]. MRNS is extremely rare and accounts for approximately 1–3% of childhood idiopathic nephrotic syndrome cases. Patients with MRNS continue to exhibit significant proteinuria and marked edema, often requiring long-term hospitalization. Moreover, attention should be paid to the development of hypertension, severe infections, acute kidney injury, bacterial peritonitis, and thromboembolisms. However, once complete remission is achieved, progression to CKD stage 5 may be avoided.

For patients with MRNS, repeated steroid pulse therapy, low-density lipoprotein absorption, and plasma exchange therapy are considered to achieve remission; however, their effectiveness has been shown only in small-scale retrospective studies [12–14]. Currently, no effective and safe remission induction therapy has been established for MRNS. Because of its rarity, prospective studies have been rarely conducted.

Rituximab is a monoclonal antibody against CD20 expressed on the surface of B cells. It is effective against various diseases caused by B-cell abnormalities because it specifically depletes CD20-positive B cells [15]. The results of studies on the use of rituximab for SRNS treatment have been published since 2007 [16–21]. Although the 2021 Kidney Disease: Improving Global Outcomes guidelines do not mention rituximab for the treatment of SRNS, the International Pediatric Nephrology Association weakly recommends its use for MRNS treatment [1, 22]. In Japan, rituximab is currently used off-label in patients with SRNS who cannot achieve remission with standard immunosuppressant therapy [19, 23].

Therefore, we conducted this multicenter collaborative trial to evaluate the efficacy and safety of combination therapy with rituximab and steroid pulse therapy in addition to cyclosporine therapy in patients with childhood-onset MRNS who were resistant to cyclosporine and more than three courses of steroid pulse therapy.

## Patients and methods

### Study design

This was a multicenter, non-blinded, single-arm trial of rituximab for the treatment of childhood-onset MRNS. The inclusion of a placebo group in this study was considered ethically difficult because participants who have not achieved complete remission with immunosuppressive drug treatment and/or steroid pulse therapy are likely to eventually develop kidney failure. Additionally, because of the rarity of the disease, it would be difficult to accumulate the required number of participants for a comparative study. Therefore, this trial did not include a control group and was an open-label, multicenter, single-arm study. To fully evaluate efficacy and safety, we defined the treatment period as the period from day 1 (the first day of the first course of steroid pulse therapy) to day 169 of the intervention with the combination therapy. The follow-up period was set from the day after the treatment period to the end of the trial (2.5 years after the trial initiation).

### Implementation

The investigators explained the contents of this clinical trial to potential study participants and/or their guardians, who provided written consent for participation. Thereafter, a screening examination was performed to verify participant eligibility. Patients whose eligibility was verified were registered to the trial within 35 days of obtaining consent.

### Study participants

Patients were included if they met all of the following inclusion criteria:

Idiopathic nephrotic syndrome (based on the diagnostic criteria of the International Study of Kidney Disease in Children [ISKDC] at initial diagnosis [4]);
Age < 18 years at the onset of idiopathic nephrotic syndrome (with no upper age limit at enrollment);
MRNS that had been treated with both a) and b) below within 1 year before enrollment:
  - A calcineurin inhibitor (cyclosporine or tacrolimus) for at least 2 months within 4 months of registration;
  - Steroid pulse therapy (3–12 sessions);
  However, patients who had undergone three or more sessions of steroid pulse therapy within 1 year before enrollment and had been treated with a calcineurin inhibitor (cyclosporine or tacrolimus) for at least 1 month who still required at least 6 days of albumin infusion per week to manage edema and were unable to continue treatment with both a) and b) were considered eligible for inclusion in this study.
Urinary protein-to-urinary creatinine ratio (Up/Uc) of ≥ 2.0 g/g creatinine (Cr) and serum albumin level of ≤ 2.5 g/dL in both of the two measurements conducted within 4 weeks before enrollment (the second measurement date must be > 7 days after the first measurement date);
(5) At least five CD20-positive cells per microliter in peripheral blood (in facilities where the number of CD20-positive cells cannot be measured, CD19-positive cells can be counted instead).

Patients were excluded if they met any of the following exclusion criteria:

1. A diagnosis of a nephritic condition (e.g., IgA nephropathy) or suspicion of a secondary nephrotic syndrome;
2. Presence or suspicion of a genetic abnormality associated with the onset of nephrotic syndrome. Before registration, all patients underwent a comprehensive gene analysis through next-generation sequencing using a custom-targeted sequencing panel for 78 genes previously reported to be associated with nephrotic syndrome [24]. All patients with positive gene screening results were excluded from this study.
3. Use of new immunosuppressive drugs (including high-dose glucocorticoid therapy) within 4 weeks before registration or use of an increased dose of immunosuppressants or prednisolone within 4 weeks before registration (however, an increase in calcineurin inhibitor dose for blood concentration adjustment was allowed);
4. Any of the following infections:
  - Severe infections (e.g., pneumonia and pyelonephritis) that required hospitalization or a history of infection within 6 months before registration;
  - An opportunistic infection (e.g., cytomegalovirus infection, systemic fungal infection, *Pneumocystis* infection, and non-tuberculous mycobacterial infection) or a history of these infections within 6 months before registration;
  - Active tuberculosis;
  - Suspicion of latent tuberculosis;
  - Active hepatitis B virus (HBV) or hepatitis C virus (HCV) infection or a confirmed HBV carrier state;
  - Human immunodeficiency virus (HIV) infection;
5. Angina pectoris, heart failure, myocardial infarction, or advanced arrhythmia;
6. Live vaccination within 4 weeks before registration;
7. Uncontrolled hypertension despite treatment with antihypertensive drugs at the time of enrollment;
8. Autoimmune diseases, IgA vasculitis, or a history of these conditions;
9. Current malignancy or a history of malignancy;
10. Organ transplantation (except corneal or hair transplantation.);
11. A history of drug allergy to methylprednisolone, acetaminophen, or d-chlorpheniramine maleate;
12. Kidney dysfunction (estimated glomerular filtration rate [eGFR] < 45 mL/min/1.73 m^2^) at enrollment;
13. Any one of the following clinical examination items at the time of registration (evaluated within 2 weeks before registration):
  - White blood cells: < 3000/µL;
  - Neutrophils: < 1500/µL;
  - Platelets: < 50,000/µL;
  - Aspartate aminotransferase (serum glutamic oxaloacetic transaminase): at least 2.5 times the upper limit of the reference value;
  - Alanine aminotransferase (serum glutamic pyruvic transaminase): at least 2.5 times the upper limit of the reference value;
  - Hepatitis B surface (HBs) antigen, HBs antibody, hepatitis B core antibody, HCV antibody: positive for any (however, among patients positive for only HBs antibody and with a history of HBV vaccination, those with negative HBV DNA quantification result at the time of enrollment were considered eligible);
  - HIV antibody: positive.
14. Use of monoclonal antibodies other than rituximab (mouse, rat, chimera, or human);
15. History of rituximab use within 1 year before registration;
16. Treatment with other clinical trial drugs within 6 months before enrollment or planned participation in other investigations during the study period;
17. Refusal to use contraception during the treatment period in patients in whom pregnancy was possible (confirmation with a serum human chorionic gonadotropin test was mandatory at screening);
18. Confirmed pregnancy, possible pregnancy, or a nursing status in female patients;
19. Ineligibility for other reasons as judged by a physician.

### Intervention

**Fig. 1:**
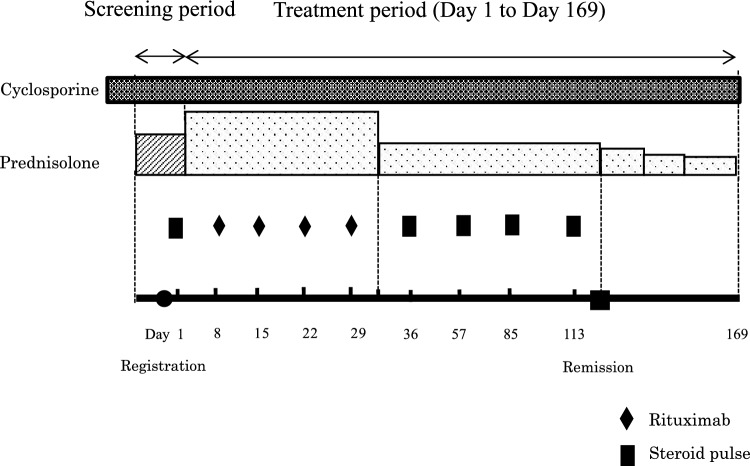
Study regimen. The investigators administered the first dose of steroid pulse therapy within 14 days after the date of registration (the first day of the first course of steroid pulse therapy was set as day 1). Rituximab was administered in four 375 mg/m^2^ doses (maximum dose: 500 mg), separated by 1 week (days 8, 15, 22, and 29). The prednisolone tapering plan was as follows: (1) 30 mg/m^2^/day (maximum dose: 30 mg/day) divided into three portions for 4 weeks; (2) 30 mg/m^2^/dose (maximum dose: 30 mg/dose) once every other day until urine protein negativity was confirmed for three consecutive days; (3) 25 mg/m^2^/dose (maximum dose: 25 mg/dose) once every other day for 4 weeks; (4) 20 mg/m^2^/dose (maximum dose: 20 mg/dose) once every other day for 4 weeks; (5) 15 mg/m^2^/dose (maximum dose: 15 mg/dose) continued once every other day

Steroid pulse therapy consisted of one course of intravenous methylprednisolone succinate sodium 30 mg/kg/day (maximum dose: 1000 mg/day) administered over 1–2 h for three consecutive days. The first dose of the first course of steroid pulse therapy was administered within 14 days of the registration date (the date of administration of the first dose of steroid pulse therapy was set as day 1, week 1). Rituximab was administered from week 2 in four 375 mg/m^2^ doses (maximum dose: 500 mg) at weekly intervals (8, 15, 22, and 29 days), followed by a maximum of four courses of additional steroid pulse therapy until remission was achieved (five courses in total). Participants who achieved remission before completing the fifth course of steroid pulse therapy received no further courses after the last steroid pulse therapy. Prednisolone was maintained with tapering doses until the end of the treatment period (Fig. 1). Premedication consisting of acetaminophen, d-chlorpheniramine maleate, and methylprednisolone was administered 30 min before the rituximab infusion to decrease the incidence and severity of infusion reactions. Prednisolone treatment for relapse during the treatment period was administered following the ISKDC regimen [2]. To prevent *Pneumocystis carinii* infection, we administered sulfamethoxazole/trimethoprim until normalization of the number of B cells in peripheral blood.

Combination therapy with the following drugs and treatments was prohibited during the treatment period: (1) commercial rituximab; (2) immunosuppressants or alkylating agents with an immunosuppressive effect, unless cases were determined to be treatment failures; (3) low-density lipoprotein adsorbents for direct hemoperfusion; (4) plasma exchange; (5) angiotensin-converting enzyme inhibitors and angiotensin receptor blockers newly started for purposes other than hypertension treatment; (6) live vaccines; and (7) other investigational drugs or unapproved drugs in Japan. Albumin preparations were administered during the treatment period if clinically necessary. The trial schedule is presented in Supplementary Table 1.

No treatment restrictions were applied during the follow-up period.

### Outcomes

The primary endpoint was a > 50% reduction in Up/Uc from baseline on day 169. The primary endpoint was set to confirm efficacy equivalent to the achievement of incomplete remission in each case, considering the number of patients in this study, the number of patients requiring albumin administration, and the variability of Up/Uc at baseline.

The secondary endpoints were set as follows: (1) number and proportion of patients achieving a > 50% reduction in Up/Uc from baseline on day 169, with Up/Uc of 0.2–2.0 g/g Cr; (2) number and proportion of patients achieving complete remission, defined as negative for early morning urine protein (assessed using the test paper method) for three consecutive days or early morning urine with a Up/Uc of < 0.2 g/g Cr for three consecutive days, on day 169; (3) number and proportion of patients achieving incomplete remission, defined as early morning urine protein ≥ 1 + (assessed using the test paper method) or early morning urine showing a Up/Uc of ≥ 0.2 g/g Cr and serum albumin > 2.5 g/dL, on day 169; (4) total number and proportion of patients achieving remission (incomplete or complete) on day 169; (5) number and proportion of patients showing a nephrotic status on day 169; (6) number and proportion of patients who progressed to chronic kidney failure, defined as eGFR < 45 mL/min/1.73 m^2^ sustained for 3 months, during the treatment period; (7) time to incomplete remission; (8) time to complete remission; (9) period of peripheral blood B-cell depletion, defined as < 5 cells/µL; and (10) changes in Up/Uc, eGFR, and serum albumin levels during the study.

Two other outcomes were analyzed: the blood concentration of rituximab and the production of human anti-chimeric antibodies (HACAs) (Supplementary Table 1).

Adverse events and adverse reactions, defined as adverse events judged to be related to the study drug (including unknown related substances), during the treatment and follow-up periods were analyzed as safety outcomes.

### Statistical methods

#### Sample size

The planned number of participants was five.

We did not determine the number of participants based on any statistical inference because the targeted patients for this study are extremely rare. Approximately 1–3% of the patients were expected to satisfy the eligibility criteria of this study, and the number of patients that can be enrolled was assumed to be approximately 5–15 per year. Considering the feasibility of the study period, we estimated the maximum number of participants to be enrolled in the study to be five.

#### Efficacy evaluation

Proportions with 95% confidence intervals were estimated for the primary endpoint, remission status, nephrotic status, and development of chronic kidney failure. Medians with 95% confidence intervals of time to remission and recovery of peripheral blood B cells were estimated using the Kaplan–Meier method.

Because the planned sample size was only five, which was not sufficient for statistical inference, we did not set any success criterion for the primary endpoint based on statistical inference. We focused on assessing the changes in the observed Up/Uc values in each participant rather than summarizing any statistical measure.

#### Data analysis for other outcomes

For the blood concentration of rituximab, pharmacokinetic parameters such as the area under the curve (AUC, µg h/mL) and maximum concentration (*C*_max_, ng/mL) were estimated. For HACAs, proportions and cumulative production rates with 95% confidence intervals were estimated.

## Results

Between June 1, 2019, and September 30, 2021, six patients were enrolled in this trial and received the combination therapy. Five of the six patients completed the follow-up period, whereas one patient discontinued participation at the end of the treatment period because of difficulty in visiting the hospital. All six patients were included in the primary analysis set. The participants’ characteristics are shown in Table 1.

**Table 1:** Participants’ characteristics

Number of participants	–	6 (100.0%)
Sex	Male	2 (33.3%)
	Female	4 (66.7%)
Age (years)	Mean (sd)	9.0 (5.8)
	Median [min, max]	8.5 [1, 19]
Ethnicity	Asian	6 (100.0%)
Height (cm)	Mean (sd)	123.80 (27.67)
(at the time of enrollment)	Median [min, max]	130.55 [76.5, 157.0]
Weight (kg)	Mean (sd)	32.45 (21.11)
(at the time of enrollment)	Median [min, max]	28.30 [9.4, 72.1]
History of calcineurin inhibitor treatment within 4 months prior to enrollment	Cyclosporine Tacrolimus Cyclosporine/Tacrolimus	5 (83.3%) 0 (0.0%) 1 (16.7%)
Number of steroid pulse treatments within 1 year before enrollment	3 courses 5 courses 6 courses	3 (50.0%) 2 (33.3%) 1 (16.7%)
Albumin administration for at least 6 days per week at the time of enrollment	–	1 (16.7%)
Previous administration of rituximab	–	1 (16.7%)
Renal biopsy histology findings	Minimal change	1 (16.7%)
	Focal segmental glomerulosclerosis Mesangium proliferation	4 (66.7%) 1 (16.7%)
Pre-existing medical history	–	3 (50.0%)
Any complicating disorder	–	6 (100.0%)
On ACE inhibitor medication	–	3 (50.0%)
On ARB inhibitor medication	–	2 (33.3%)

*ACE* angiotensin-converting enzyme inhibitor, *ARB* angiotensin II receptor blocker

## Compliancing

All six participants received four 375 mg/m^2^ doses of rituximab. Four participants completed five courses of steroid pulse therapy, whereas one participant received only two courses and one participant received only four courses because they had already achieved complete remission.

## Effectiveness results

**Fig. 2:**
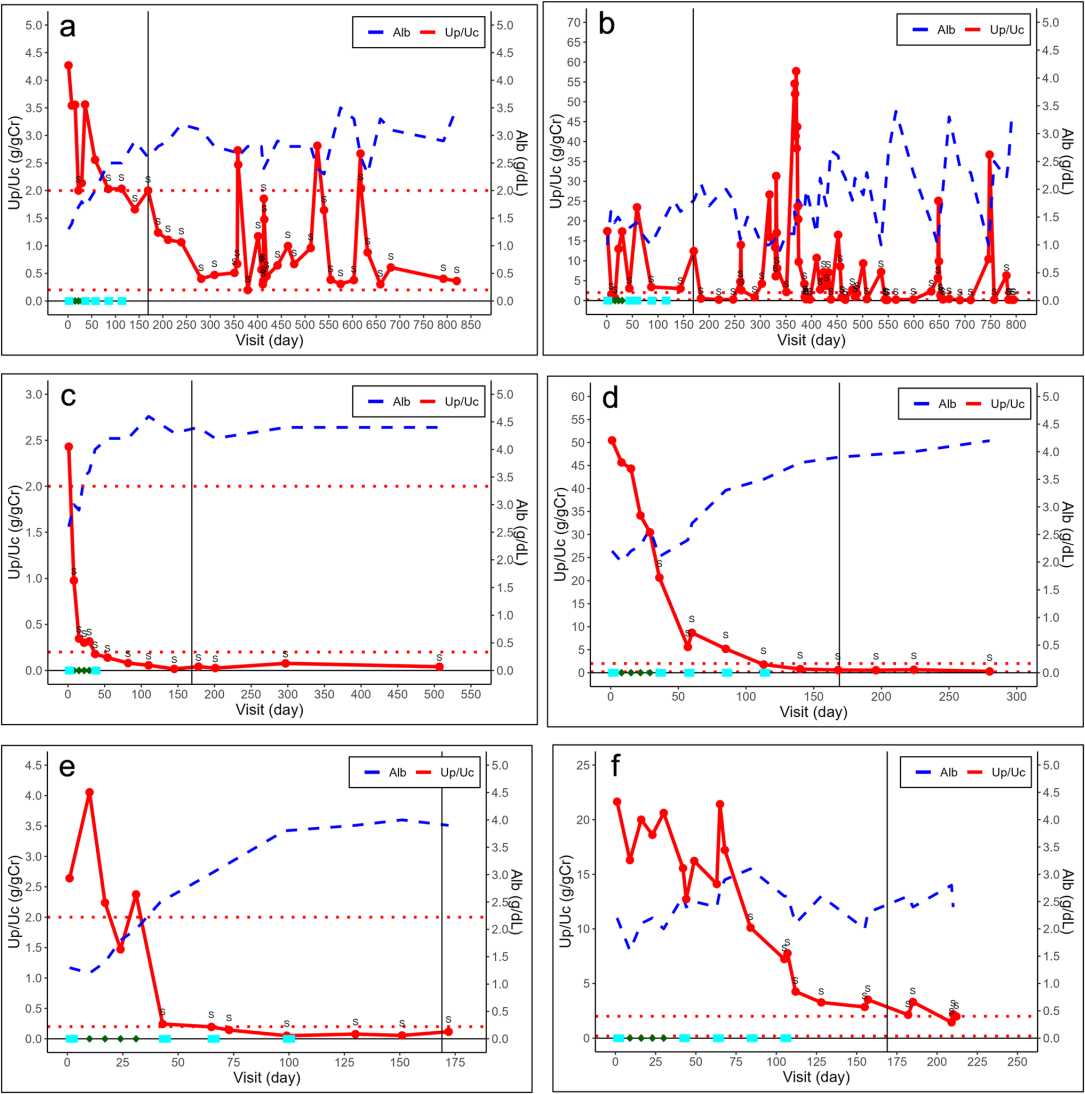
Changes in urinary protein-to-urinary creatinine ratio and serum albumin levels. The graphs (**a–f**) show the urinary protein-to-urinary creatinine ratio (solid line) and the serum albumin levels (dashed line) during the treatment and follow-up periods in each participant, cases 1–6, respectively. “S” means attainment of a > 50% reduction in the urinary protein-to-urinary creatinine ratio from baseline. The first dose of the first course of steroid pulse therapy was administered on day 1. Rituximab was administered in four 375 mg/m^2^ doses at weekly intervals (8, 15, 22, and 29 days), followed by a maximum of four courses (dark green diamonds) of additional steroid pulse therapy (five courses in total, cyan squares). The reference line on the horizontal axis represents day 169, which was the end of the treatment period. Up/Uc, urinary protein-to-urinary creatinine ratio; Cr, creatinine; Alb, albumin

**Fig. 3:**
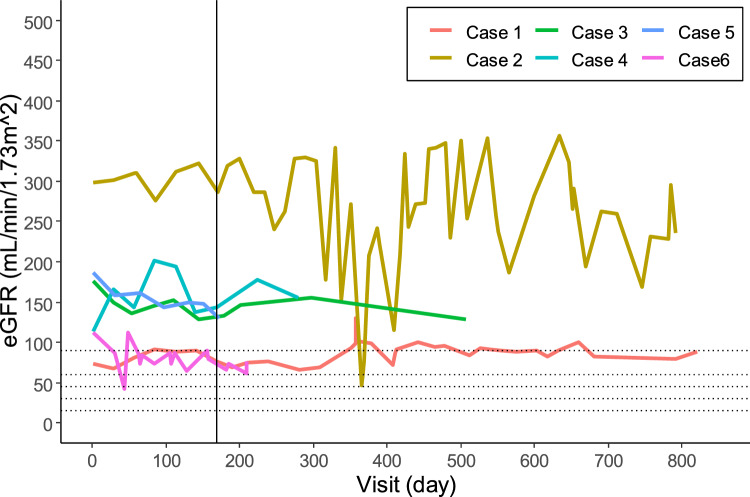
Changes in estimated glomerular filtration rate (eGFR). The graph shows the eGFR values during the treatment and follow-up periods in each participant

Of the six participants, five achieved the primary endpoint. The proportions of participants who achieved each endpoint are summarized in Table 2. As one participant achieved partial remission on day 1, the number of participants in the analysis set for time to incomplete remission (a secondary endpoint) was five instead of six (Table 3). Figure 2 shows the changes in Up/Uc and serum albumin levels over time in each participant. Changes in eGFR are shown in Fig. 3. A detailed description of each participant is provided in Supplementary Data 1.

**Table 2:** Proportions of the primary endpoint, remission, nephrotic status, and chronic renal failure development

	Numbers in analysis set	Achieved number	Proportion (%)	95% confidence interval [%]
(Primary endpoint) 50% reduction of the Up/Uc at Day 169	6	5	83.3	[43.6, 97.0]
(1) More than 50% reduction of the Up/Uc and the Up/Uc is 0.2 to 2.0 g/gCr at Day 169	6	2	33.3	[9.7, 70.0]
(2) Complete remission at Day 169	6	2	33.3	[9.7, 70.0]
(3) Incomplete remission at Day 169	6	2	33.3	[9.7, 70.0]
(4) Either incomplete or complete remission at Day 169	6	4	66.7	[30.0, 90.3]
(5) Nephrotic status at Day 169	6	2	33.3	[9.7, 70.0]
(6) Chronic renal failure development during the treatment period	6	0	0.0	[0.0, 39.0]

*Up/Uc* urinary protein creatinine ratio

**Table 3:** Median time to remission and recovery of peripheral blood B cell

	Numbers in analysis set	Median [days]	95% confidence interval [days]
(7) time to incomplete remission	5	133.0	[52.0, –]
(8) time to complete remission	6	371.0	[29.0, –]
(9) time to recovering of peripheral blood B cell	5	112.0	[31.0, –]

The primary endpoint (> 50% reduction in Up/Uc from baseline on day 169) was observed in five of the six participants (83.3%). In the participant who did not achieve the primary endpoint, the rate of decrease in Up/Uc from baseline was 28.8% on day 169.

Regarding the rituximab blood concentration, the mean ± standard deviation (SD) values of the AUC and C_max_ in the six participants were 171,000 ± 85,100 μg h/mL and 300,000 ± 58,300 ng/mL, respectively. The mean ± SD values of the half-life (in the central compartment model), clearance, mean retention time, and volume of distribution were 78.8 ± 58.5 h, 0.0192 ± 0.0164 L/h, 114 ± 84.3 h, and 1.57 ± 0.626 L, respectively.

Three (50.0%), two (33.3%), and four (66.7%) participants showed HACA positivity at visits 2, 8, and 11 (day 169), respectively. The cumulative HACA production rates at each visit according to the Kaplan–Meier method were 50.0%, 66.7%, and 100.0%, respectively.

One of the six participants discontinued trial participation after the treatment period, and five participants were evaluated for the remission status and transition to chronic kidney failure at the time of follow-up. Of these, four participants (80.0%) achieved incomplete remission or better at the end of follow-up (two were in partial remission and two were in complete remission). One participant (20.0%) was nephrotic at the end of follow-up. None of the participants transitioned to chronic kidney failure during the follow-up.

## Safety evaluation

Forty-one adverse events occurred in the six participants. Of these, 21 adverse events (in five of the six participants) were judged to be related to the study drug. Dyspnea and oropharyngeal discomfort occurred as adverse reactions in two participants each, and other adverse reactions were observed in the remaining two participants. Seven adverse events in four patients were judged to be associated with steroid pulse therapy: fever in two patients and nasopharyngitis, dysgeusia, taste disorder, hypertension, and acne in one patient each.

No deaths occurred. Serious adverse events occurred in two patients (two instances of infection, both in case 2; three instances of acute kidney injury, all in case 6), which were resolved within several days. These adverse events were judged as serious because they required admission or an extended stay in a hospital or clinic for treatment.

Six infections requiring treatment occurred in three patients, including one case of grade 3 infection and one case of impetigo. Despite the median time for peripheral blood B cell recovery being 112 days (as indicated in Table 3), it is noteworthy that three out of the six reported adverse events of moderate severity transpired during the period of B-cell depletion.

Fourteen mild or moderate infusion reaction events occurred in five patients, which resolved within the day of onset.

In this study, there were no instances of persistent hypogammaglobulinemia observed among the participants.

## Discussion

This clinical trial was conducted to assess the efficacy and safety of rituximab in combination with steroid pulse therapy and standard cyclosporine therapy in patients with childhood-onset MRNS. Patients who do not achieve complete remission after immunosuppressive drug treatment are likely to eventually develop kidney failure. In small-scale studies, the effectiveness of plasma exchange or low-density lipoprotein (LDL) absorption for managing MRNS has been demonstrated [25]. Nevertheless, these treatment modalities are invasive and pose challenges in their application to pediatric patients. Additionally, their implementation is constrained to specialized facilities. Furthermore, the therapeutic outcomes yielded by these methods have not proven to be entirely satisfactory.

Our team previously conducted a multicenter, double-blind, placebo-controlled, randomized controlled trial that confirmed the efficacy of rituximab in maintaining remission for a longer period in patients with childhood-onset intractable nephrotic syndrome [26]. Based on the trial results, the use of rituximab in the treatment of complicated, frequently relapsing/steroid-dependent nephrotic syndrome was approved in Japan in 2014.

The use of rituximab for SRNS treatment has been reported since 2007 [16, 19, 21, 27]. In a multicenter cohort study, rituximab was administered four times at weekly intervals to 33 patients with SRNS; the remission rate at 6 months was 48.5% (16/33 cases; complete remission in 9 cases, incomplete remission in 7 cases), the complete remission rate was 27.3% (9/33 cases), and the median time from the last dose of rituximab to the induction of remission was 32 days (8–60 days) [17]. Moreover, in another multicenter cohort study, rituximab was administered to 19 patients with SRNS. The remission rate was 63.2% (12/19 cases; complete remission in 6 cases, incomplete remission in 6 cases), and the complete remission rate was 31.6% (6/19 cases). The time from the last day of rituximab administration to the start of the remission period was 5.1 ± 3.1 months (6 months, 1–12 months) [18]. In a randomized controlled trial of two doses of rituximab (375 mg/m^2^/dose, maximum dose: 500 mg/dose) administered to 31 patients with SRNS, the effectiveness of rituximab was not verified based on the rate of change in proteinuria levels [20]. In a retrospective observational study of rituximab administration (two doses of 375 mg/m^2^/dose) and steroid pulse therapy (every 2–4 weeks until complete remission) in 10 patients with SRNS, 7 patients achieved complete remission (6 cases of complete remission within 6 months of rituximab administration), 1 patient achieved partial remission, and this approach was ineffective in 2 patients. During the observation period (median observation period: 35 months), patients who achieved complete remission were able to maintain normal kidney function; however, the patient in partial remission had a mild decline in kidney function and the two patients in whom the approach was ineffective progressed to CKD stage 5 [19]. The current situation regarding the treatment of this condition has been well-reviewed by Kamei et al. [28].

In this study, we targeted MRNS cases that are resistant to cyclosporine and steroid pulse therapy, the most severe form of SRNS, and evaluated the effect of rituximab. The combination therapy used in this trial attained a remission rate of 66.7% (four of six cases; two cases of complete remission, two cases of partial remission). At the planning stage, we assumed that the primary endpoint will be achieved in 27.3% of the participants, which is similar to the lower value of complete remission rate reported in previous studies [16, 21, 27]. The primary endpoint was achieved in 83.3% (five of six) of our participants (confidence interval: 43.6%–97.0%), which is a much higher rate than we expected.

When it comes to assessing the safety of rituximab treatment for refractory nephrotic syndrome, recent extensive cohort marketing surveillance data have unequivocally established its safety, as indicated by reference [29]. Furthermore, a recent retrospective cohort study conducted at a single center has put forth the possibility that rituximab treatment could potentially result in sustained disease remission over the long term in individuals with refractory nephrotic syndrome, as alluded to in reference [30].

We considered the combination therapy safe and tolerable, as all serious adverse events (infection and acute kidney injury) were known and resolved with treatment. Infusion reactions specific to rituximab also resolved on the day of onset in all participants.

## Limitations

The limitations of this trial were the small sample size and the lack of an internal control group. Owing to the rare nature of the disease and the ethical difficulty of including a placebo group, only a single-arm trial was feasible. Because of the small sample size, the study was designed to confirm the effect of the therapy based on a statistical test. However, we expected the proportion of patients achieving the primary endpoint to be higher than the proportion of patients achieving complete or partial remission. Therefore, we considered that a sample of five patients is sufficient to evaluate the efficacy and safety of rituximab for the target disease.

## Conclusion

The combination of rituximab and steroid pulse therapy in addition to baseline cyclosporine was effective in reducing Up/Uc and showed remission-inducing effects in patients with childhood-onset MRNS. The incidence of adverse events in this trial was within the expected range; therefore, this treatment method has excellent safety and tolerability. However, infection prevention measures are vital during periods of peripheral blood B-cell depletion.

## Participating hospitals

Dokkyo Medical University Hospital, Japan, Nihon University Itabashi Hospital, Japan, National Center for Child Health and Development, Japan, Tokyo Metropolitan Children’s Medical Center, Japan, Yokohama City University Medical Center, Japan, Shiga University of Medical Science Hospital, Japan, Kobe University Graduate School of Medicine, Japan, Hyogo Prefectural Kobe Children’s Hospital, Japan, Wakayama Medical University, Japan, Kurume University Hospital, Japan, Saga University Hospital, Japan.

## Data sharing plan

Will individual deidentified participant data (including data dictionaries) be available? Yes.

Which data in particular will be shared? Individual participant data that underlie the results reported in this article, after deidentification (text, tables, figures, and appendices).

Will additional, related documents be available? Yes. Study Protocol and Statistical Analysis Plan.

When will the data become available and for how long? Beginning 3 months and ending 5 years after article publication.

By what access criteria will the data be shared? Proposals should be directed to the clinical trial secretariat (nozu@med.kobe-u.ac.jp). To gain access, those requesting data will need to sign a data access agreement.

## Supplementary Information

Below is the link to the electronic supplementary material.

Supplementary file1 (DOCX 38 KB)

Supplementary file2 (XLSX 16 KB)

## Data Availability

The datasets generated and/or analyzed in the current study are available from the corresponding author upon reasonable request.
