# Supplementary material for: Rituximab in combination with cyclosporine and steroid pulse therapy for childhood-onset multidrug-resistant nephrotic syndrome: a multicenter single-arm clinical trial (JSKDC11 trial)
Source: Clin Exp Nephrol. 2023 Nov 27;28(4):337–48. doi: 10.1007/s10157-023-02431-0 (PMC10955017; doi:10.1007/s10157-023-02431-0)
Supplement: Supplementary file 1 — Supplementary file1 (DOCX 38 KB) [file 10157_2023_2431_MOESM1_ESM.docx]

**Supplementary Data 1**

***Participant descriptions***

**Case 1 (Age: 8 years 1 month girl, height: 111.3 cm, weight: 24.9 kg, pathological finding: mesangial proliferation)**

Steroid pulse therapy was administered at a dose of 570 mg/dose for three consecutive days, for a total of five courses. Rituximab was administered at 290 mg/dose every week for a total of four doses.

The Up/Uc during the treatment period was 4.27 g/gCr at baseline and 2.00 g/gCr on Day 169, with a 53.2% reduction. On Day 169, the participant attained incomplete remission. Entering the follow-up period, complete remission was attained and the time to complete remission was 371 days. Thereafter, two relapses were identified during the follow-up period, both of which led to incomplete remission within four weeks after the start of daily prednisolone treatment. At the end of the follow-up period, the patient was in incomplete remission with an Up/Uc of 0.36 g/gCr (Fig. 2a).

The eGFR was 73.1 mL/min/1.73 m^2^ at baseline, with no apparent decline during the treatment period, and 76.5 mL/min/1.73 m^2^ at Day 169. There was also no apparent decline during the follow-up period, and at the end of the follow-up period, it was 88.5 mL/min/1.73 m^2^ (Fig. 3).

Peripheral blood B-cell counts were depleted at 29 days and recovered at 141 days. The duration of B-cell depletion in peripheral blood was 112 days.

Seven adverse events occurred. The infusion reaction was hypertension in the second dose of rituximab; the rate of rituximab administration was slowed down due to the onset of the infusion reaction, but the patient recovered 15 minutes after the onset and the full prescribed dose was completed.

**Case 2 (Age: 1 year 9 months, girl, height: 76.5 cm, weight: 9.4 kg, pathological finding: focal segmental glomerulonephritis)**

Steroid pulse therapy in this trial was administered at 279 mg/dose for three consecutive days for a total of five courses, but only on day 3 of the third course due to fever. Rituximab was administered at 160 mg/dose every week for a total of four doses.

The Up/Uc during the treatment period was 17.45 g/gCr at baseline and 12.44 g/gCr on Day 169, with a decrease of 28.8%. The participant was in a nephrotic state on Day 169, but at the start of the follow-up period, incomplete remission and complete remission were observed. The time to incomplete remission was 429 days and to complete remission 538 days. Two relapses were subsequently identified during the follow-up period and steroid pulse therapy was administered as a treatment for relapse. At the end of the follow-up period, the patient was in complete remission (Fig. 2b).

The eGFR was 298.9 mL/min/1.73 m^2^ at baseline, with no apparent decline during the treatment period, and 285.8 mL/min/1.73 m^2^ at Day 169. There was also a temporary drop to 46.6 mL/min/1.73m^2^ during the follow-up period, which subsequently recovered and was 235.1 mL/min/1.73m^2^ at the end of the follow-up period (Fig. 3).

The number of B cells in the peripheral blood decreased to 27 cells/µL at 30 days but increased thereafter and no depletion was observed.

Six adverse events occurred. Vomiting (first dose of rituximab) occurred as an infusion reaction (first dose of rituximab). Rituximab administration was temporarily interrupted due to the onset of an infusion reaction, and the participant subsequently recovered. After recovery, administration of the study drug was resumed and the full prescribed dose was completed. Adverse events other than infusion reaction included two infections. Both two infections were judged to be serious, requiring hospitalization or prolonged hospitalization in a hospital for treatment, and were judged to be related to rituximab. The participant recovered within a few days of onset.

**Case 3 (Age: 9 years 10 months, girl, height: 136.4 cm, weight: 32.4 kg, pathological finding: minimal change)**

Steroid pulse therapy was administered at 975 mg/dose for three consecutive days, for a total of two courses. Rituximab was administered at 420 mg/dose every week for a total of four doses.

The Up/Uc during the treatment period was 2.43 g/gCr at baseline and 0.04 g/gCr on Day 169, with a 98.3% reduction. The participant had the Up/Uc of 2.43 g/gCr and serum albumin of 2.6 g/dL on Day 1 and was therefore attained to be in incomplete remission prior to the start of the rituximab. Therefore, the time to incomplete remission could not be calculated; A Up/Uc of 0.18 g/gCr was achieved at 37 days, attaining a complete remission. The time to complete remission was 29 days; complete remission was maintained from Day 169 to the end of the follow-up period (Fig. 2c).

The eGFR was 176.7 mL/min/1.73 m^2^ at baseline, with no apparent decline during the treatment period, and 132.9 mL/min/1.73 m^2^ at Day 169. There was also no apparent decline during the follow-up period, with 128.5 mL/min/1.73m^2^ at the end of the follow-up period (Fig. 3).

Peripheral blood B-cell counts were depleted at 29 days and recovered at 178 days.

Four adverse events occurred. Infusion reaction occurred in three cases: two cases of dyspnoea and one case of oropharyngeal discomfort (both at the first dose of the study drug). After recovery, administration of rituximab was resumed and the full prescribed dose was completed.

**Case 4 (Age: 7 years 1 month, boy, height: 124.7 cm, weight: 24.2 kg, pathological finding: focal segmental glomerulonephritis)**

Due to an error in the height used to calculate the dose of the steroid pulse, steroid pulse therapy was administered at 700 mg on day 1 of the first course, 740 mg on days 2 and 3 of the first course and on day 1 of the second course; from day 2 of the second course onwards, the prescribed dose was 770 mg/dose, for a total of five courses. Rituximab was administered at 360 mg/dose every week for a total of four doses.

The Up/Uc during the treatment period was 50.46 g/gCr at baseline and 0.58 g/gCr at Day 169, with a 98.9% reduction. During the treatment period, the Up/Uc was 8.69 g/gCr and serum albumin 2.7 g/dL, attaining incomplete remission. The time to incomplete remission was 52 days. At the end of the follow-up period, the Up/Uc was 0.29 g/gCr and the participant remained in incomplete remission from Day 169 to the end of the follow-up period (Fig. 2d).

The eGFR was 113.5 mL/min/1.73 m^2^ at baseline, with no apparent decline during the treatment period, and 142.9 mL/min/1.73 m^2^ at Day 169. There was also no apparent decline during the follow-up period, with a value of 154.7 mL/min/1.73m^2^ at the end of the follow-up period (Fig. 3).

Peripheral blood B-cell counts were depleted at 113 days and recovered at 168 days The duration of B-cell depletion in peripheral blood was 55 days.

Nine adverse events occurred. Five infusion reactions occurred, one each of cough, abdominal pain, dyspnea, nasal obstruction, and rhinorrhea (all on the first dose of the study drug). Rituximab administration was not changed, but recovery was achieved after 35 minutes. After recovery, rituximab was resumed and the full prescribed dose was completed.

**Case 5 (Age: 10 years 4 month, boy, height: 136.9 cm, weight: 31.7 kg, pathological finding: focal segmental glomerulonephritis)**

Steroid pulse therapy was administered at 1000 mg/dose for three consecutive days, for a total of four courses. Rituximab was administered at 420 mg/dose every week for a total of four doses. The participant was discontinued the trial after completing the treatment period because the participant could not come to the hospital due to reasons (busy schedule, relocation, hospital transfer, etc.).

The Up/Uc during the treatment period was 2.64 g/gCr at baseline and 0.12 g/gCr on Day 169, with a 95.6% reduction. During the treatment period day 57, the Up/Uc was 0.20 g/gCr and serum albumin 3.0 g/dL, attaining incomplete remission. At 73 days, the Up/Uc was 0.14 g/gCr, attaining a complete remission. Time to incomplete remission and time to complete remission were 55 and 63 days, respectively; at Day 169, the participant remained in complete remission (Fig 2e).

eGFR was 187.2 mL/min/1.73 m^2^ at baseline, with no apparent decline during the treatment period, and 129.4 mL/min/1.73 m^2^ at Day 169 (Fig. 3).

Peripheral blood B-cell counts were depleted at 99 days and recovered at 130 days. The duration of B-cell depletion in peripheral blood was 31 days. Peripheral blood B-cell counts were depleted again at 151 days and recovered at 172 days.

Five adverse events occurred. No infusion reactions were observed.

**Case 6 (Age 19 years 0 months, female, height 157.0 cm, weight 72.1 kg)**

Steroid pulse therapy was administered at 1000 mg/dose for three consecutive days, for a total of five courses. Rituximab was administered at 500 mg/dose every week for a total of four doses.

The Up/Uc during the treatment period was 21.64 g/gCr at baseline and 2.85 g/gCr on Day 169, with a reduction of 86.8%. The participant was nephrotic throughout the treatment and follow-up periods. At the end of the follow-up period, the Up/Uc was 1.99 g/gCr (Fig 2f).

The eGFR was 111.8 mL/min/1.73 m^2^ at baseline and temporarily decreased to 42.6 mL/min/1.73 m^2^ due to acute renal failure associated with the primary disease during the treatment period, but subsequently recovered and was 89.6 mL/min/1.73 m^2^ at Day 169. At the end of the follow-up period, it was 76.5 mL/min/1.73m^2^ (Fig. 3).

Peripheral blood B-cell counts were depleted at 30 days, with no recovery observed during the treatment period.

Ten adverse events occurred. Infusion reactions occurred in all four administrations, all of which were oropharyngeal discomfort. There was no reduction in the rate of rituximab administration or interruption of rituximab administration, and the participant recovered within a few hours after the onset of symptoms. As adverse events other than infusion reactions, three acute kidney injuries were judged to be serious, but these adverse events disappeared within a few days of onset.
